# Ketogenic metabolic therapy as a candidate adjunct for CBTp delivery in schizophrenia spectrum disorders

**DOI:** 10.3389/fpsyg.2026.1775511

**Published:** 2026-03-25

**Authors:** Nicole Laurent

**Affiliations:** Family Renewal, Inc., Vancouver, WA, United States

**Keywords:** schizophrenia spectrum disorders, psychosis, cognitive behavioral therapy for psychosis, CBTp, ketogenic metabolic therapy, ketogenic diet, metabolic psychiatry, treatment engagement

## Abstract

Schizophrenia spectrum disorders (SSDs) are managed with pharmacotherapy alongside psychosocial interventions, including cognitive-behavioral therapy for psychosis (CBTp). In routine services, uptake and completion of CBTp remain limited, and participation barriers disrupt initiation, retention, and between-session follow-through. Ketogenic metabolic therapy (KMT) is a dietary intervention intended to induce and maintain nutritional ketosis through carbohydrate restriction and a higher fat dietary pattern, with education, monitoring, and safety management integrated into care. Early reports utilizing KMT in SSDs describe improvements in domains that limit CBTp delivery, including sleep disruption, arousal and distress reactivity, mood instability, cognitive burden, and day to day functioning, while effects on positive symptoms remain uncertain and evidence is methodologically heterogeneous. This perspective article situates KMT within CBTp engagement frameworks and reframes an actionable question for clinical psychologists: whether KMT can reduce participation limiting factors enough to increase CBTp initiation, retention, and completion when psychotherapy is truncated by instability, cognitive burden, distress, or practical barriers. A care pathway model is outlined that organizes participation barriers across entry into therapy and continuity across transitions back to community care, with CBTp indicators proposed for evaluating adjunct impact. If future trials support this adjunct role, KMT may represent a practical strategy for increasing CBTp dose and strengthening access to recovery-oriented gains in coping, stability, and functional outcomes, even when positive symptoms persist or fluctuate. These elements form a clinically grounded conceptual model that specifies measurable indicators and testable decision points for evaluating KMT as an adjunct to CBTp delivery in routine care.

## 1 Introduction

Schizophrenia spectrum disorders (SSD) are commonly managed with antipsychotic pharmacotherapy alongside psychosocial interventions, with cognitive behavioral therapy for psychosis (CBTp) and family intervention positioned as standard psychological components of comprehensive care in major treatment guidelines and clinical pathways (Mueser et al., 2015; Galletly et al., 2016; Keepers et al., 2020). Coordinated specialty care models for first episode psychosis emphasize early engagement and recovery oriented, goal focused treatment planning (Dixon et al., 2015; Mueser et al., 2015). These models prioritize functional outcomes such as education, employment, and social relationships through structured psychosocial components delivered by multidisciplinary teams (Dixon et al., 2015; Mueser et al., 2015; Kane et al., 2016). In this context, the clinically actionable question for clinical psychologists is not whether CBTp is theoretically indicated, but whether individuals can engage in and sustain sufficient participation for CBTp to be delivered as intended and translated into meaningful functional change (Villeneuve et al., 2010; Fahy et al., 2025; Wood et al., 2025).

Evidence syntheses support CBTp as an intervention with small to medium effects on psychotic symptoms, with effects varying by symptom domain and study characteristics (Turner et al., 2014; Sitko et al., 2020; Berendsen et al., 2024; Tan et al., 2026). These effects are most clinically meaningful when CBTp is received and when participation barriers do not truncate treatment prematurely (Sitko et al., 2020; Bouchard et al., 2022), Engagement barriers in first episode psychosis psychotherapy have been described across patient, family, service, and system levels, including factors that delay entry into care, reduce retention, and limit uptake of structured psychological work (Mueser et al., 2015; Fahy et al., 2025). Dropout from psychosocial treatment is also a measurable phenomenon in SSD, and it varies by setting and study characteristics, suggesting that treatment participation cannot be assumed even when psychotherapy is offered (Villeneuve et al., 2010).

Despite guideline recommendations and an established evidence base (Tan et al., 2026), receipt of psychological therapy for psychosis and schizophrenia remains low internationally, and implementation studies continue to document limited delivery of CBTp and other recommended psychological interventions in routine services (Taylor and Perera, 2015; Colling et al., 2017; Burgess-Barr et al., 2023, 2024). This implementation gap matters clinically because it concentrates risk in the same subgroup that is already vulnerable to poor functional outcomes, repeated crises, and disrupted developmental trajectories, meaning those who struggle to stay engaged long enough to benefit from psychosocial care (Mueser et al., 2015; Fahy et al., 2025). Within a clinical psychology frame, limited participation is best treated as a capacity and context problem rather than a simple refusal of treatment, because participation is shaped by symptom burden, cognitive load, stress reactivity, family environment, and service accessibility (Galletly et al., 2016; Fahy et al., 2025).

This conceptual analysis uses an integrative narrative review and conceptual synthesis focused on CBTp delivery in routine care from a clinical psychology perspective. In this context, “psychiatry” refers to the routine care pathway and multidisciplinary medical setting in which psychosis is managed and in which CBTp is offered, rather than to a single etiologic model. Evidence describing CBTp engagement and retention barriers across care pathways were synthesized with available Ketogenic Metabolic Therapy (KMT) evidence relevant to symptom and functional domains that commonly constrain psychotherapy participation. These bodies of work were then integrated by mapping participation barriers to candidate KMT associated outcome domains, with evaluation anchored to CBTp initiation, session continuity, and between session follow through rather than disorder level efficacy. This synthesis is hypothesis generating and did not use systematic review methods.

## 2 Positioning KMT within CBTp engagement frameworks

Metabolic psychiatry is an emerging field focused on the intersection of metabolism and mental health that investigates bidirectional mechanisms between metabolism and mental health to inform new or improved interventions for psychiatric disorders (Mudra Rakshasa-Loots et al., 2025). Within this framework KMT is increasingly being studied as a metabolic treatment (Sethi and Ford, 2022; Rog et al., 2024; Mudra Rakshasa-Loots et al., 2025). KMT, also known as the ketogenic diet (KD), refers to clinician supervised ketogenic dietary interventions intended to induce and maintain nutritional ketosis through carbohydrate restriction and a higher fat dietary pattern, with structured education, monitoring, and safety management integrated into care (Sethi and Ford, 2022; Rog et al., 2024). Structured clinical considerations for KMT delivery have been clarified through a Delphi consensus providing guidance on KMT implementation and safety management in clinical contexts (Ede et al., 2026), as well as an exploration of its relevance for clinical psychologists (Laurent et al., 2024). Ketogenic dietary therapies have an established clinical history in drug resistant epilepsy, where guidance emphasizes protocol specification, monitoring, and proactive management of adverse effects and contraindications as core determinants of tolerability and continuation (Martin-McGill et al., 2020; Kossoff et al., 2018). In metabolic psychiatry, KMT is increasingly framed as a potentially transdiagnostic intervention for serious mental illness, with hypothesized relevance to brain energy metabolism, oxidative stress, inflammation, and neurotransmitter systems (Sethi and Ford, 2022; Anderson et al., 2024; Rog et al., 2024; Hung et al., 2025), including emerging immunometabolic mechanisms in which ketone bodies such as β hydroxybutyrate may influence inflammatory signaling and gene regulation (Hu et al., 2025), and explored within the clinical psychology literature in terms of common mechanisms relevant to multiple disorders (Laurent et al., 2024).

The evidence base for KMT as a treatment for SSD remains methodologically heterogeneous, particularly in case reports and uncontrolled series where dietary formulations, verification of ketosis, implementation supports, and follow up vary substantially. In contrast, controlled trials in serious mental illness are emerging and tend to be more standardized (Chrysafi et al., 2024; Rog et al., 2024). These limitations support a cautious interpretation of symptom change reports and clarify the need for standardized protocols, prespecified outcomes, and clear safety procedures that can be implemented in real world services (Chrysafi et al., 2024; Longhitano et al., 2024; Rog et al., 2024). Within this context, there are examples of ketogenic diet implementation and symptom assessment in schizophrenia spectrum populations, and psychiatric outcomes reported during KMT in SSD include findings that may have relevance to CBTp engagement (Table 1).

**Table 1:** Psychiatric outcomes reported during KMT in schizophrenia-spectrum disorders: relevance to CBTp engagement.

**Study/design**	**Participant(s)**	**Measurement and timeline**	**Key findings relevant to CBTp engagement**
(Pacheco et al. 1965)/inpatient pilot study	Ten female inpatients with chronic schizophrenia (ages 19–63; nine undifferentiated, one paranoid)	Beckomberga rating scale (S-factor): baseline (first 2 days) → 2 weeks on diet → 1 week after discontinuation; average score decreased significantly at 2 weeks; 7/10 showed increased symptomatology at 1-week post-discontinuation, remaining improved vs. baseline	Reduced symptom burden over 2 weeks; increased symptomatology after discontinuation; residual improvement vs. baseline
(Palmer 2017)/case series; case 1	33-year-old man with schizoaffective disorder	PANSS: 98 (P 27, N 25, G 46) at baseline (Jan 2016) → 49 (P 13, N 8, G 28) at ~1 year	Reduced positive and negative symptoms; reported improvements in mood, energy, and concentration; functional gains (education participation, social engagement, independent living); symptom worsening with dietary interruption and improvement after resumption
(Palmer 2017)/case series; case 2	31-year-old woman with schizoaffective disorder	PANSS: 107 (P 24, N 29, G 54) at baseline (Mar 2016) → 70 (P 15, N 18, G 38) at ~4 months	Psychotic symptoms: reduced positive and negative symptoms; delusions reported to remit within weeks; reported improvements in mood and energy; symptom recurrence after discontinuation and subsequent rapid resolution reported after resumption/brief fast
(Palmer et al. 2019)/case series; patient A	82-year-old woman with schizophrenia	Psychotic symptoms: marked reduction ≤ 2 weeks after diet initiation; complete remission reported thereafter; long-term follow-up reported (12 years). PANSS assessed pre/post; values not reported	Marked reduction of psychosis within 2 weeks, complete remission, and 12 year follow up without medications, regained independent living
(Palmer et al. 2019)/case series; patient B	39-year-old woman with schizophrenia	Psychotic symptoms: complete resolution reported within 1 month of diet initiation; remission maintained off antipsychotic medication at 5-year follow-up. PANSS assessed pre/post; values not reported	Reduced psychotic symptoms burden with complete remission; sustained remission off antipsychotic medication at 5 year follow up; restored role functioning
(Danan et al. 2022)/retrospective inpatient series	Ten adults with schizoaffective disorder in the analyzed cohort (*n* = 28)	Pre/post inpatient intervention: PANSS 91.4 → 49.3 (*n* = 10); CGI-S 5.4 → 2.6 (*n* = 9); HAM-D 28.2 → 7.0 (*n* = 5); MADRS 32.7 → 9.3 (*n* = 3); CGI-S improved in all assessed patients	Reduced psychosis symptom burden with clinically meaningful change in all PANSS-assessed schizoaffective participants; reduced depressive symptom burden; reduced global illness severity. Marked reductions in psychosis severity and global illness severity; reported minimal clinically important difference on PANSS achieved in 10/10 assessed patients; concurrent reductions in depressive symptom burden
(Sethi et al. 2024)/single-arm pilot trial	Five adults with schizophrenia-spectrum disorders (schizophrenia *n* = 2; schizoaffective disorder *n* = 3) within a 21-participant pilot cohort	BPRS (baseline mean 44.0 ± 20.2; 32% reduction at 4 months; schizophrenia-spectrum subgroup); CGI severity of mental illness (baseline 3.6 ± 1.0; change −1.3 ± 1.3 at 4 months; full cohort); GAF (baseline mean 63.8 ± 8.0; 17% improvement at 4 months; full cohort); MANSA quality of life (baseline mean 4.3 ± 1.0; change +0.6 ± 0.7 at 4 months; full cohort); PSQI sleep disturbance (baseline mean 7.9 ± 3.7; change −1.7 ± 2.8 at 4 months; full cohort)	Reduced psychotic symptoms burden; improved global illness severity; improved functioning and quality of life; improved sleep
(Laurent et al. 2025)/retrospective case series; case 1	17-year-old female with schizoaffective disorder	Suicidal ideation, depression/anxiety, and hallucinations reported fully resolved by 6 weeks; GAD-7 total 8 → 1 (baseline, 6, 19, 24 weeks); DASS-42 total 48 → 14 (baseline, 6, 19, 24 weeks); PCL-5 total 38 → 8 (baseline, 6, 19, 24 weeks)	Rapid remission of hallucinations and suicidal ideation; reduced anxiety/depression/stress and trauma-related distress; return to school/work
(Laurent et al. 2025)/retrospective case series; case 2	32-year-old female with schizoaffective disorder	Psychotic symptoms reported markedly reduced beginning ~2 weeks after initiation; GAD-7 total 6 → 0 (baseline, 8, 13, 22, 27, 52 weeks); DASS-42 total 15 → 3 (baseline, 8, 13, 22, 27, 52 weeks); PCL-5 total 8 → 3 (baseline, 8, 13, 22, 27, 52 weeks); PHQ-9 total 3 → 0 (baseline, 8, 13, 22, 27, 52 weeks)	Early reduction in psychotic symptom interference (with hallucinations described as resolving early); reported improvement in cognitive symptoms over time; sustained reductions in anxiety/depressive symptoms across follow-up
(Murray et al. 2025)/case report	48-year-old woman with treatment-resistant schizophrenia; inpatient	Pre to post 5 weeks, Simpson Angus Scale 15 → 3. Brief Psychiatric Rating Scale total 43 → 48, negative symptoms 10 → 8, positive symptoms 6 → 7	Marked reduction in extrapyramidal side effects, with modest improvement in negative symptoms, could reduce participation limiting physical discomfort and psychomotor slowing
Newiss (2025)/case report	32-year-old male with schizophrenia	No validated psychiatric symptom rating scales reported. Remission noted by mental health team after 6-months KMT followed by community treatment order discharge	Marked reduction in extrapyramidal side effects, with modest reduction in negative symptoms

This table summarizes schizophrenia-spectrum case reports/series and early clinical studies reporting psychiatric symptoms and functioning outcomes during KMT, with key findings summarized as indicators relevant to CBTp engagement.

An early inpatient pilot administered a ketogenic diet to 10 female patients with chronic schizophrenia and assessed change using the Beckomberga Rating Scale (S-Factor) during the first 2 study days, after 2 weeks on the diet, and 1 week after discontinuation Pacheco et al., (1965). Average Beckomberga scores decreased after 2 weeks and increased in most patients 1 week after discontinuation, while remaining improved relative to baseline Pacheco et al., (1965).

In a two case series of schizoaffective disorder, a 33-year-old man with persistent positive and negative symptoms initiated a ketogenic diet. Psychosis severity was reported to decrease over follow up, with auditory hallucinations and delusions reported to lessen within 3 weeks, alongside concurrent improvements in mood, energy, concentration, and functioning Palmer, (2017). In the same series, a 31-year-old woman with schizoaffective disorder initiated a ketogenic diet. Delusions were reported to remit within 4 weeks, recur after discontinuation, and then resolve again after resumption of the ketogenic diet and a brief fasting period, alongside improvement in overall psychosis severity over follow up Palmer, (2017).

In another case series of schizophrenia, an 82-year-old woman with schizophrenia since age 17 and chronic daily hallucinations and paranoia initiated a ketogenic diet. Marked symptom reduction was reported within 2 weeks, and sustained remission was described over long term follow up off antipsychotic medication, alongside functional change from reliance on a PACT team and a court-appointed guardian to independent living Palmer et al., (2019). In the same case series, a 39-year-old woman with long standing hallucinations and paranoia continued a ketogenic diet during a subsequent hospitalization after abrupt discontinuation of psychiatric medications. Symptom resolution was reported within 1 month, with sustained remission described for 5 years off antipsychotic medication, alongside functional recovery including completion of graduate school and full-time employment Palmer et al., (2019).

A retrospective inpatient series reported large pre-to-post reductions in psychosis symptom severity on the Positive and Negative Syndrome Scale (PANSS) in the schizoaffective subgroup (*n* = 10), with all assessed patients meeting a clinically meaningful reduction threshold Danan et al., (2022). In the same subgroup, global illness severity improved on the Clinical Global Impressions Severity scale (CGI-S), and depressive symptom measures improved in the assessed subsets. Across the overall inpatient sample the authors reported broad improvement in global illness severity on the CGI-S, including improvement in nearly all assessed patients Danan et al., (2022).

A retrospective case series described a 17-year-old female with schizoaffective disorder and severe suicidal ideation with auditory and visual hallucinations who initiated KMT with medical oversight. Resolution of suicidal ideation and hallucinations was reported by 6 weeks, with sustained improvement in self-reported symptoms through 24 weeks, alongside return to studies and work Laurent et al., (2025). In the same series, a 32-year-old female with schizoaffective disorder, psychotic episodes, and prominent cognitive symptoms initiated KMT with medical oversight. Marked reduction in psychotic symptoms was reported within approximately 2 weeks, with longitudinal improvement in mood and distress measures tracked through 52 weeks Laurent et al., (2025).

In a 4-month pilot trial, schizophrenia spectrum participants received an outpatient ketogenic diet intervention alongside usual psychiatric care, with structured medical monitoring and midpoint and final psychiatric assessments (Sethi et al., 2024). For schizophrenia outcomes, the study reported a 32% reduction in Brief Psychiatric Rating Scale (BPRS) total score from baseline to study end. By study end, all adherent participants were classified as recovered, and 33% of semi-adherent participants achieved recovery during the study, with improvements reported in overall function, life satisfaction, and sleep (Sethi et al., 2024).

In a retrospective case report, a 32-year-old man with schizophrenia initiated a carnivore ketogenic diet after discharge from a rehabilitation hospital with nutritional therapy practitioner support (Newiss, 2025). The report described remission noted by his mental health team with no further psychotic episodes over more than 9 months, alongside tapering and discontinuation of psychiatric medications and subsequent discharge of a community treatment order, with some ongoing low mood attributed to socio economic circumstances (Newiss, 2025).

In a 5-week inpatient case report, a 48-year-old woman with treatment-resistant schizophrenia and metabolic syndrome completed a medically supervised ketogenic diet without antipsychotic dose changes (Murray et al., 2025). Although global psychopathology ratings did not improve at the endpoint the authors described a marked reduction in antipsychotic-associated extrapyramidal symptoms and a modest improvement in negative symptoms, and the participant reported the diet made a positive change and expressed interest in continuing (Murray et al., 2025).

Notably, the domains most often described as limiting CBTp uptake and between session practice, including distress reactivity, sleep disruption, mood instability, cognitive burden, and day to day functioning (Fahy et al., 2025), are also prerequisites for core CBTp processes such as collaborative formulation and between session follow through (Morrison and Barratt, 2010; Berry and Hayward, 2011). Qualitative evidence synthesis in first episode psychosis has shown that engagement is hindered by emotional distress and fluctuating symptoms, and that therapy could be experienced as too cognitively demanding due to concentration difficulties (Fahy et al., 2025). The same synthesis reported that efforts toward improved sleep and experiencing early positive changes in symptoms were viewed as useful and encouraged engagement (Fahy et al., 2025). These domains, most commonly implicated in limiting CBTp engagement and supporting therapeutic gains, overlap with those most consistently reported to improve with KMT in schizophrenia spectrum presentations (Sethi and Ford, 2022; Rog et al., 2024).

**Figure 1:**
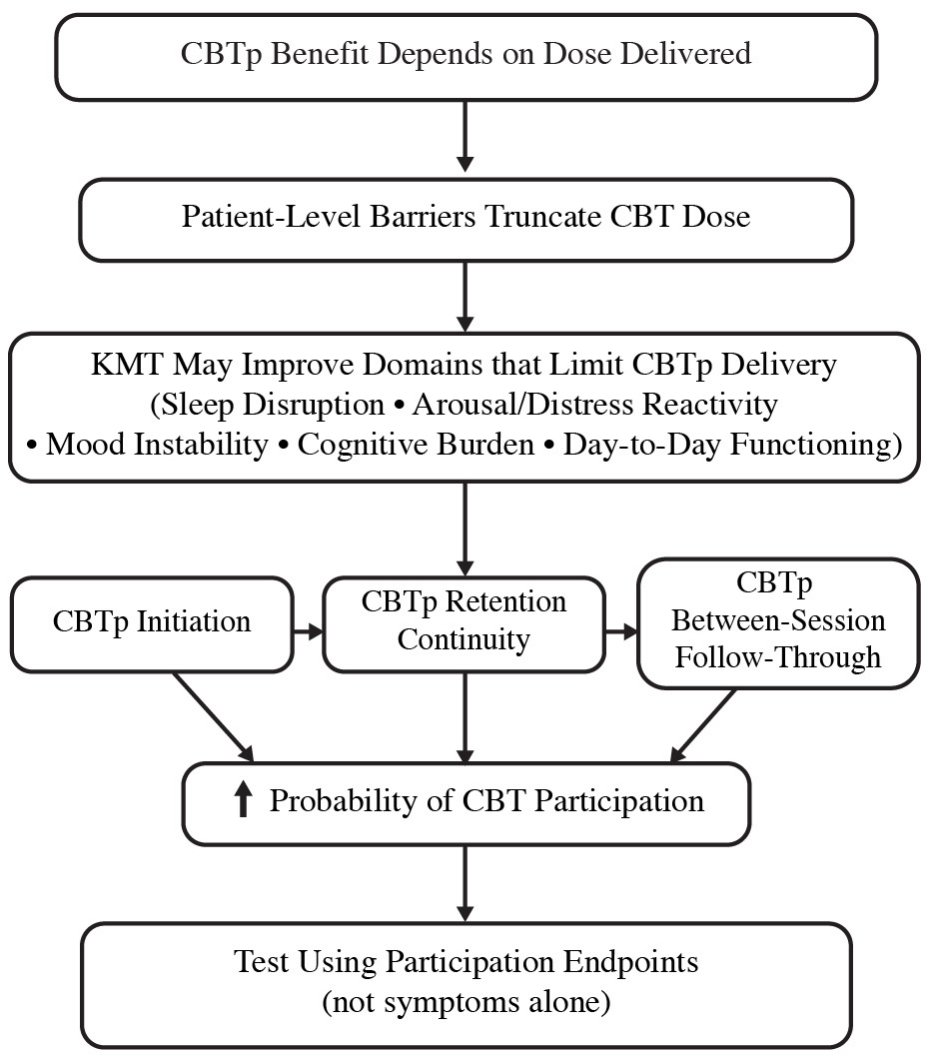
KMT participation-capacity framework for patient-level barriers to CBTp delivery in schizophrenia spectrum disorders. This conceptual diagram illustrates a hypothesized pathway through which ketogenic metabolic therapy (KMT) may reduce common patient-level barriers (e.g., sleep disruption, cognitive burden, mood instability) that limit participation in cognitive-behavioral therapy for psychosis (CBTp). It emphasizes evaluating KMT effectiveness using clearly defined CBTp participation endpoints rather than symptom reduction alone.

This evidence base does not justify broad conclusions that KMT treats schizophrenia spectrum disorders (Sethi and Ford, 2022; Rog et al., 2024). It does justify a narrower and clinically actionable question aligned with psychotherapy delivery: whether KMT can improve participation limiting factors enough to increase CBTp initiation, retention, and completion when CBTp is otherwise truncated by instability, cognitive burden, distress, or practical barriers (Berry and Hayward, 2011; Rog et al., 2024). Figure 1 summarizes a participation-capacity framework illustrating how KMT-associated improvements in these domains may enhance CBTp initiation, session continuity, and between-session follow-through.

## 3 Clinical, functional, and psychosocial indicators that shape CBTp participation

Treatment participation in CBTp extends beyond treatment initiation. It includes uptake, sustained attendance, and a working alliance that supports collaborative psychological work over time (Berry and Hayward, 2011; Fahy et al., 2025; Wood et al., 2025). This distinction is clinically relevant because CBTp includes identifiable components that depend on continued collaboration, pacing, and follow through rather than brief exposure to treatment (Morrison and Barratt, 2010; Wood et al., 2022; Konings et al., 2025). When participation becomes inconsistent or truncated, the primary clinical question is often whether participation limiting conditions can be reduced sufficiently for CBTp to be delivered as intended (Villeneuve et al., 2010; Fahy et al., 2025).

Engagement in CBTp is shaped by clinical state and perceived safety of disclosure, including distress and risk burden, symptom fluctuation, negative symptoms, paranoia, cognitive capacity, and stigma related concerns about talking openly about psychotic experiences (Fahy et al., 2025). Cognitive load is relevant because CBTp is typically collaborative and active. It uses guided discovery, monitoring methods, and shared formulation work to identify targets and select change strategies, and service users describe the process as developing new understandings, coping strategies, and alternative explanations for psychotic experiences (Morrison and Barratt, 2010; Berry and Hayward, 2011; Wood et al., 2022; Konings et al., 2025). In acute inpatient or otherwise restrictive contexts, engagement may be further constrained by crisis conditions and environmental limitations, including sedation, reduced privacy, and concerns that disclosure could affect clinical decisions such as medication changes, leave, and admission length, supporting the need for flexible, patient led delivery and clear attention to autonomy and consent (Wood et al., 2022). These considerations inform routine decisions about pacing and session structure, including simplifying language, tailoring materials to memory and concentration, and using stepped or stabilization focused approaches when admission duration is brief (Morrison and Barratt, 2010; Wood et al., 2022). Across stages of therapy, barriers may manifest as non-uptake, missed sessions or refusal, or early discontinuation. When therapy proceeds, barriers may limit capacity for collaborative formulation and formulation-guided change work, including self-monitoring and between session tasks (Morrison and Barratt, 2010; Villeneuve et al., 2010; Wood et al., 2022; Fahy et al., 2025).

Functional and practical constraints can undermine sustained engagement in psychological therapy for psychosis, including regular attendance, and these factors include functional impairment (Fahy et al., 2025). Because CBT for psychosis comprises structured elements such as formulation, goals, and homework, inconsistent participation can limit exposure to planned components (Morrison and Barratt, 2010). Reported barriers include unmet practical priorities such as housing and benefits support needs, and service access constraints such as staff discontinuity, limited accessibility, and inconvenient appointment times (Fahy et al., 2025). Attrition is also common in psychosocial treatment research in psychotic disorders, and reported reasons for disengagement include being too unwell and forgetting appointments, indicating that non-completion should be anticipated when planning delivery and support (Villeneuve et al., 2010; Bouchard et al., 2022). Within CBT for psychosis, homework is intended to bridge sessions and everyday contexts, so missed appointments, reduced completion of agreed between session tasks, and early discontinuation are pragmatic indicators of reduced participation (Morrison and Barratt, 2010).

Participation in CBTp is shaped by whether therapy is experienced as safe and destigmatizing, whether the work is collaborative, and whether the therapy model and goals are aligned with the person's own understanding and priorities (Morrison and Barratt, 2010; Berry and Hayward, 2011; Fahy et al., 2025). Acceptability is supported when therapists use normalization to reduce stigma, adapt session structure and pace to the person's capacity, and support coping strategies and alternative explanations that reduce distress (Morrison and Barratt, 2010; Berry and Hayward, 2011; Fahy et al., 2025). Engagement is also influenced by service accessibility and delivery conditions, including delays and complex pathways to care, limited availability of recommended psychological interventions, and practical service factors such as staff consistency, appointment timing, and access to suitable locations (Taylor and Perera, 2015; Fahy et al., 2025; Konings et al., 2025). When fit or collaboration is poor, or when service delivery is inconsistent, participation difficulties may present as limited uptake, inconsistent attendance, or weaker working alliance, with increased risk of disengagement before the planned course is completed (Fahy et al., 2025). Measurement-based care provides a practical framework for monitoring response and engagement by using systematic standardized assessments to inform clinical decision making and guide treatment adjustments over time (Aboraya et al., 2018; Martin-Cook et al., 2021). Pragmatic monitoring targets in CBTp can include attendance and completion, brief symptom and functioning measures, structured client feedback on the therapy process, and planning and review of homework or other agreed between session activities (Morrison and Barratt, 2010; Aboraya et al., 2018; Martin-Cook et al., 2021).

## 4 Engagement barriers across CBTp care pathways and KMT as an adjunct

In CBTp trials, sessions received are frequently not recorded or not reported, and when reported, they are often fewer than sessions offered, so the dose actually delivered is often unclear (Sitko et al., 2020). Participation barriers appear at predictable stages in psychosis care pathways, spanning entry into therapy, delivery during acute admissions, and continuity across transitions back to community care (Wood et al., 2022, 2025; Fahy et al., 2025). Limited privacy in restrictive settings and fears about consequences of disclosure can constrain engagement even when willingness is present (Wood et al., 2022; Fahy et al., 2025). When CBTp begins, symptom fluctuation, cognitive burden, and practical instability can reduce attendance and shorten therapy before core CBTp processes can be delivered. Dropout is common across psychosocial interventions for SSD, supporting the expectation that incomplete participation will occur unless barriers are actively addressed (Villeneuve et al., 2010; Bouchard et al., 2022; Fahy et al., 2025). To systematically address these participation barriers and measurement gaps, clearly defined research priorities are required. Because participation measurement is often incomplete in CBTp trials, Table 2 specifies research priorities and participation endpoints for testing KMT as a candidate adjunct to CBTp delivery in SSD.

**Table 2:** Research priorities for KMT as a candidate adjunct to CBTp participation in schizophrenia-spectrum disorders.

**Priority/decision point**	**Participation endpoints to report**	**Feasibility and acceptability indicators**	**Design and reporting priorities**	**Relevant categories of assessment**	**Research rationale and key sources**
Engagement failure, non-initiation or early discontinuation	CBTp initiation rate, % referred attending ≥1 session, time from referral to first CBTp session, early discontinuation timing, dropout before session X, delivered dose, sessions offered, sessions attended, attendance frequency, % completing prespecified minimally adequate course, core component dose, formulation work and homework planning and review documented	Patient rated helpfulness and acceptability, reasons for non-initiation or dropout, brief structured items plus optional qualitative, alliance and collaboration indicators when continuity is the evaluation target, perceived burden and pace fit, including therapy feeling overwhelming or emotionally distressing, KMT tolerability and KMT discontinuation reasons tracked separately from CBTp discontinuation	Prespecify participation endpoints as the adjunct target, define CBTp dose by core components including homework planning and review, prespecify KMT sequencing before or alongside early CBTp engagement when testing initiation and early retention, prespecify KMT target domains that commonly limit CBTp entry, sleep disruption, arousal and distress reactivity, mood instability, cognitive burden, day-to-day functioning; if change, then test whether change in these domains coincided with formulation work and between-session practice	Alliance and engagement, expectancy and satisfaction, implementation acceptability and feasibility, attrition reasons and barriers, participation limiting domains targeted by KMT paired with initiation and early continuity endpoints	CBTp is guideline recommended for schizophrenia, so participation outcomes should quantify delivered dose rather than assume exposure. CBT showed small to medium end treatment effects in SSD with weaker or inconsistent follow up effects, supporting dose and continuity measurement when participation is the evaluation target (Colling et al., 2017; Keepers et al., 2020; Sitko et al., 2020; Berendsen et al., 2024)
Between session practice failure despite continued contact	Homework completion rate, % planned tasks completed, homework review frequency, % sessions with planned review completed, treatment enactment indicators, self-monitoring frequency and strategy use between sessions	Patient reported cognitive load and concentration difficulties affecting between-session follow-through, patient reported distress-related interference with completing between-session work when present, perceived relevance and burden of between-session tasks	Define between-session practice expectations within the CBTp model being evaluated, measure practice failure separately from attendance failure, prespecify KMT sequencing as concurrent with CBTp or introduced after early CBTp pacing and adaptation when homework follow-through is the participation target, prespecify KMT target domains most relevant to practice completion, cognitive burden, sleep disruption, distress reactivity, mood instability, day-to-day functioning. If change is observed in these domains, then test for associated increases in homework completion and enactment	Homework completion and review, treatment enactment and adherence, barriers to follow-through tied to cognitive load and distress, participation limiting domains targeted by KMT paired with homework and enactment endpoints	CBTp standards include formulation and homework planning and review as core elements, and trials frequently omit adherence reporting, so delivered dose should include between session practice rather than attendance only (Morrison and Barratt, 2010; Berry and Hayward, 2011; Sitko et al., 2020)
Context constrained continuity, service and social instability	Missed session rate and reschedule count when practical barriers dominate, continuity across service transitions and changes in service intensity, CBTp continuation during periods of instability or competing care demands when present	Service limitations and unmet therapy preference when present, physical capacity constraints when present, access barriers, transport, scheduling, referral delays, clinician availability, KMT feasibility within routine services, monitoring burden and supervision access	Report care setting and pathway context, including coordinated specialty care vs. community outpatient delivery when relevant, document concurrent psychosocial components that affect engagement and continuity, prespecify whether KMT is tested as an adjunct for participation limited by instability, stress reactivity, cognitive burden, and reduced day-to-day functioning rather than as a symptom-focused intervention, measure whether KMT-related change in these domains coincided with improved session continuity and fewer disruptions while also documenting whether KMT delivery demands introduced additional participation burden	Service access and service limitations, context linked discontinuation reasons, engagement barriers tied to capacity and distress, participation limiting domains targeted by KMT paired with continuity endpoints	Engagement barriers in early psychosis include service limitations and physical capacity constraints. APA recommends assertive community treatment when poor engagement leads to relapse or social disruption, so participation outcomes should be interpreted within service and contextual constraints (Colling et al., 2017; Keepers et al., 2020; Fahy et al., 2025)
KMT implementation fidelity, ketosis verification and monitoring	KMT initiation rate and continuation over the CBTp evaluation window, ketosis verification method and frequency, adherence indicators, tracking and monitoring completion, safety outcomes and lab monitoring plan, AE monitoring, and reasons for KMT discontinuation	Patient rated tolerability and burden of dietary change and monitoring, feasibility constraints affecting staged or combined KMT and CBTp participation, monitoring demands, supervision access, impact of KMT monitoring burden on CBTp attendance and continuity	Specify KMT protocol, screening, contraindication management, monitoring plan, and supervision model, report ketosis verification and safety monitoring procedures, prespecify how KMT monitoring contacts and CBTp sessions are scheduled to protect psychotherapy access when delivered dose is a primary endpoint	Diet formulation, adherence and ketosis confirmation, safety monitoring and AE reporting, implementation intensity measures that may affect psychotherapy participation	Safety monitoring and AE reporting, implementation intensity measures that may affect psychotherapy participation, and documentation of ketosis verification and monitoring procedures are central to adjunct interpretability because KMT risks and monitoring requirements can shape feasibility and continuation, and physician and dietitian oversight is recommended in serious mental illness (Sethi and Ford, 2022; Rog et al., 2024; Ede et al., 2026)
Care transition continuity, pathway stage and return to community care	CBTp retention and completion across transitions from coordinated specialty care to less intensive services when relevant, continuity of planned CBTp components across changes in care exposure and support intensity, continuation after discharge and return to community care when relevant to the delivery context	Acceptability of continuing CBTp and KMT after discharge when routines and supports change, continuity of KMT monitoring and support after discharge when KMT is tested as an adjunct for CBTp continuation, transition related reasons for discontinuation of CBTp and or KMT	Define care pathway stage and follow-up timeframe, report service intensity changes that affect CBTp access, prespecify KMT sequencing during post-discharge return to community care when testing retention and completion, prespecify KMT target domains linked to continuity risk at this stage, sleep disruption, distress reactivity, mood instability, cognitive burden, day-to-day functioning, then test whether change in these domains coincided with improved CBTp retention, completion, and CBTp relevant outcomes	Retention and completion in coordinated specialty care contexts, continuity across pathway stages and service transitions, participation limiting domains targeted by KMT paired with retention and completion endpoints	APA recommends coordinated specialty care for first episode psychosis and CBTp for schizophrenia spectrum disorders. Coordinated specialty care programs are multicomponent and vary in intensity over time, so participation outcomes should be interpreted relative to pathway stage and service transitions that affect psychotherapy access (Mueser et al., 2015; Keepers et al., 2020)

This table outlines research priorities for evaluating ketogenic metabolic therapy (KMT) as a candidate adjunct to cognitive behavioral therapy for psychosis (CBTp) delivery in SSD. It uses psychotherapy-defined participation endpoints to specify assessment targets for quantifying delivered CBTp dose, including initiation, session continuity, and between-session follow-through. It also identifies contextual participation barriers, KMT implementation indicators including ketosis verification, and timing considerations for interpreting participation outcomes within guideline-consistent care. AE, adverse event; APA, American Psychiatric Association; CBT, cognitive behavioral therapy; CBTp, cognitive behavioral therapy for psychosis; KMT, ketogenic metabolic therapy; SMI, serious mental illness; SSD, schizophrenia spectrum disorder.

KMT can be positioned as a candidate adjunct (Chrysafi et al., 2024; Rog et al., 2024) when CBTp has been offered and adapted, yet participation remains insufficient for collaborative formulation work and between session practice. The relevant clinical psychology question is not whether KMT is a standalone psychosis treatment, but whether it can improve participation limiting domains enough to allow CBTp to be initiated, tolerated, and completed.

When participation barriers persist despite routine CBTp adaptations, the case formulation shifts from symptom focus to a delivery problem. The question becomes which participation limiting domains are dominant at the current pathway checkpoint, and whether they can be modified enough for CBTp to proceed with collaborative formulation, guided discovery, and between session practice rather than truncated contact (Morrison and Barratt, 2010; Berry and Hayward, 2011; Fahy et al., 2025). This framing also clarifies why the adjunct question remains clinically coherent even when psychotic symptoms are unchanged or fluctuate, because CBTp remains indicated for distress, coping, avoidance, demoralization, and functional recovery goals that depend on sustained participation (Mueser et al., 2015; Keepers et al., 2020; Wood et al., 2022).

## 5 Positioning KMT as an adjunct within CBTp delivery pathways

Consideration of KMT as a candidate adjunct for CBTp may be best initiated and delivered with reference to what is described in the CBTp engagement literature. When psychological therapies are recommended but not received at scale, the clinically actionable question becomes whether participation limiting conditions can be modified enough for CBTp to be initiated, sustained, and completed as intended. This question is clinically meaningful in SSD care because routine services continue to show a large gap between guideline recommendations and actual receipt of CBTp and other recommended psychological therapies (Colling et al., 2017; Keepers et al., 2020; Burgess-Barr et al., 2023). Within this frame, clinicians and researchers can examine both whether KMT can provide adjunct support for CBTp delivery and outcomes for service users, and when in the care pathway this support is most relevant given the dominant participation limiting domains. These participation domains, barriers to adequate CBTp dose and engagement, and the preliminary KMT findings that align with those domains are summarized in Table 3.

**Table 3:** CBTp engagement barriers and preliminary KMT findings relevant to psychotherapy delivery in schizophrenia-spectrum disorders.

**Research**	**Engagement and participation domains described**	**Barriers to adequate CBTp dose and engagement**	**Preliminary reports of KMT improvements in these identified barriers**	**Where KMT can be introduced or studied as an adjunct**
Berry and Hayward (2011), qualitative synthesis	CBTp framed as collaborative; participation described via therapy “ingredients” (understanding onset/coping, considering alternative explanations, normalization) and process shifts (acceptance, increased perceived power, reconceptualizing self as distinct from psychosis)	Preference-fit, expectations, alliance, distress, homework follow-through	Distress (Danan et al., 2022; Sethi et al., 2024; Laurent et al., 2025), hallucinations (Palmer, 2017; Palmer et al., 2019; Laurent et al., 2025), delusions (Palmer, 2017), paranoia (Palmer, 2017; Palmer et al., 2019), concentration (Palmer, 2017; Laurent et al., 2025), functioning (Palmer, 2017; Palmer et al., 2019; Sethi et al., 2024; Laurent et al., 2025), global symptomatology (Pacheco et al., 1965)	KMT can be considered when participation limiting distress or cognitive burden prevents sustained collaboration and follow through, and the clinical aim is sufficient continuity for CBTp processes to be delivered as intended
Fahy et al. (2025), review article	Engagement is defined as uptake, sustained attendance, and working alliance, with disengagement reflected in non-uptake, inconsistent attendance, or early discontinuation	Distress, symptoms, capacity, expectations, service access, alliance	Psychosis severity (Pacheco et al., 1965; Palmer, 2017; Palmer et al., 2019; Danan et al., 2022; Sethi et al., 2024; Laurent et al., 2025), hallucinations (Palmer, 2017; Palmer et al., 2019; Laurent et al., 2025), delusions (Palmer, 2017), distress (Palmer, 2017; Palmer et al., 2019; Danan et al., 2022; Sethi et al., 2024; Laurent et al., 2025), concentration (Palmer, 2017; Laurent et al., 2025), sleep disturbance (Sethi et al., 2024; Laurent et al., 2025)	KMT can be considered when CBTp is indicated but uptake and early participation remain insufficient for collaborative work, with success defined by improved initiation, early retention, and session continuity sufficient to reach formulation guided work and between session practice
Keepers et al. (2020), practice guideline	Guideline pathways recommend CBTp as a component of comprehensive schizophrenia care and recognize that poor engagement and poor engagement with services increase relapse risk and social disruption	Symptoms, functioning, retention, service access	Psychosis severity (Pacheco et al., 1965; Palmer, 2017; Palmer et al., 2019; Danan et al., 2022; Sethi et al., 2024; Laurent et al., 2025), distress (Palmer, 2017; Palmer et al., 2019; Danan et al., 2022; Sethi et al., 2024; Laurent et al., 2025), concentration/cognitive symptoms (Palmer, 2017; Laurent et al., 2025), functioning (Palmer, 2017; Palmer et al., 2019; Sethi et al., 2024; Laurent et al., 2025), sleep disturbance (Sethi et al., 2024; Laurent et al., 2025), quality of life (Sethi et al., 2024)	KMT can be considered when CBTp continuation is at risk and participation limiting conditions are the dominant barrier, with success defined by improved retention and completion and CBTp relevant outcomes such as reduced distress, improved coping and self-management, improved functioning, and reduced relapse and rehospitalization
Morrison and Barratt (2010), Delphi study	CBTp relies on sustained, collaborative sessions paced to the client, with between-session practice planned and reviewed and a planned ending for maintenance; early discontinuation risks incomplete delivery of core components	Capacity, homework follow-through, symptoms, distress, alliance	Psychosis severity (Pacheco et al., 1965; Palmer, 2017; Palmer et al., 2019; Danan et al., 2022; Sethi et al., 2024; Laurent et al., 2025), distress/affective burden (Danan et al., 2022; Sethi et al., 2024; Laurent et al., 2025), concentration/cognitive symptoms (Palmer, 2017; Laurent et al., 2025), functioning (Palmer, 2017; Palmer et al., 2019; Sethi et al., 2024; Laurent et al., 2025), sleep disturbance (Sethi et al., 2024; Laurent et al., 2025), quality of life (Sethi et al., 2024)	KMT can be considered when participation remains insufficient to complete formulation guided work and between session practice despite routine CBTp pacing and adaptation, with success defined by fuller receipt of planned CBTp components
Mueser et al. (2015), special section	Coordinated specialty care models of shared decision making that prioritize sustained engagement and recovery-oriented treatment participation; participation failure reflected in reduced retention and incomplete exposure to psychosocial components	Motivation, functioning, capacity, retention, service access	Energy (Palmer, 2017), mood (Palmer, 2017; Palmer et al., 2019; Danan et al., 2022; Laurent et al., 2025), sleep disturbance (Sethi et al., 2024; Laurent et al., 2025), functioning (Palmer, 2017; Palmer et al., 2019; Sethi et al., 2024; Laurent et al., 2025), psychosis severity (Pacheco et al., 1965; Palmer, 2017; Palmer et al., 2019; Danan et al., 2022; Sethi et al., 2024; Laurent et al., 2025)	KMT can be considered when participation limiting conditions reduce continuation in CBTp, and the clinical goal remains sustained participation sufficient to achieve CBTp related recovery outcomes

This table provides a summary of participation points described in CBTp delivery references, selected barriers to adequate CBTp dose and engagement, and preliminary KMT reports that may theoretically map onto those barriers. This table is not intended to provide an exhaustive summary of the many barriers identified to CBTp delivery and implementation identified in the literature.

One such phase is when CBTp is indicated and offered, but uptake and early participation are insufficient for collaborative CBTp work to begin or to reach formulation guided change strategies. Engagement barriers described in first episode psychosis psychotherapy include emotional distress, symptom fluctuation, cognitive load, negative expectations, and practical capacity constraints, while facilitators include a destigmatizing frame, service accessibility, and a collaborative therapist style (Fahy et al., 2025). Validated measures are available for key CBTp participation constructs, including alliance, engagement, expectancy, acceptability, feasibility, treatment integrity, and barriers to initiating and continuing CBTp (Horvath and Greenberg, 1989; Devilly and Borkovec, 2000; Haddock et al., 2001; Rollinson et al., 2008; O'Brien et al., 2009; Mohr et al., 2010; Weiner et al., 2017), and future trials of KMT as an adjunct should prespecify instruments and assessment timing so participation outcomes can be interpreted beyond retention counts (Montgomery et al., 2018). Success should be defined in CBTp endpoints, initiation after referral, reduced early discontinuation, stronger working alliance, and sufficient early session receipt to reach collaborative formulation and between-session practice (Morrison and Barratt, 2010; Berry and Hayward, 2011; Fahy et al., 2025).

**Figure 2:**
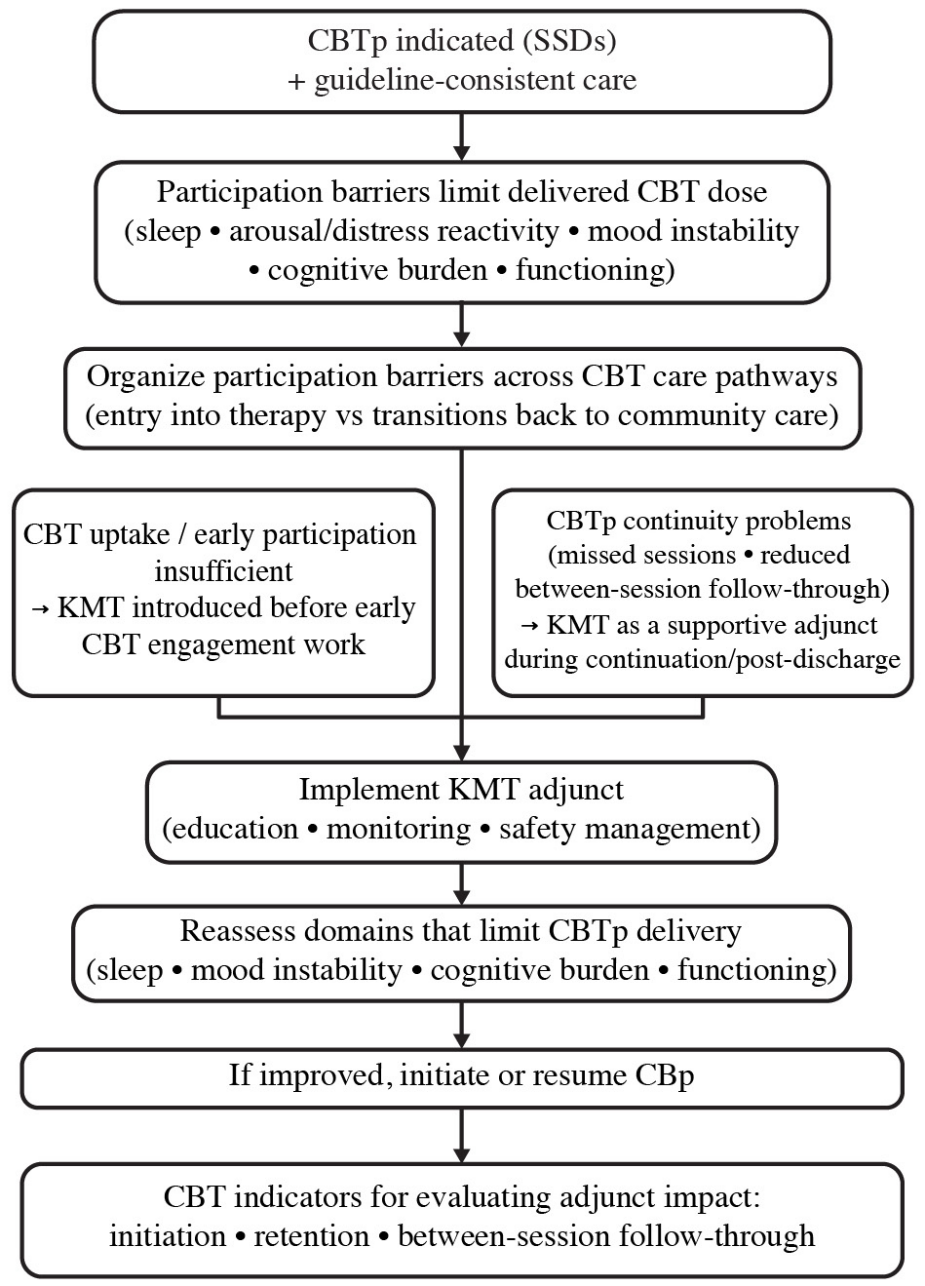
KMT participation-capacity care pathway model for participation barriers limiting CBTp delivery in schizophrenia spectrum disorders. Hypothesized clinical decision-making pathway illustrating points at which ketogenic metabolic therapy (KMT) could be introduced as a candidate adjunct to cognitive-behavioral therapy for psychosis (CBTp). The model emphasizes systematically reassessing patient-level barriers that restrict CBTp uptake, continuity, and between-session follow-through after implementing KMT. CBTp participation indicators (initiation, retention, and between-session practice), as well as feasibility and adherence indicators for KMT (education, monitoring, safety management), provide endpoints for evaluating adjunctive benefit.

KMT could be considered a supportive adjunct after discharge and during return to community care, when continuity problems commonly truncate CBTp before intended dose and recovery-oriented goals are achieved. This transition is also where service structure and engagement strategies matter most and coordinated specialty care models emphasize maintaining engagement and facilitating treatment participation through flexible contact that matches stage, needs, and preference (Dixon et al., 2015; Mueser et al., 2015). Guideline pathways also recommend CBTp as a component of comprehensive schizophrenia care, and escalate service intensity when engagement failures contribute to relapse risk and functional disruption, reinforcing that non-participation is treated as a pathway problem with structured service responses (Keepers et al., 2020). KMT, integrated as an adjunct during the post discharge phase when CBTp continuation is at risk, may increase CBTp retention and completion, and possibly improve CBTp relevant outcomes. Assessment may evaluate instances of reduced distress, improved coping and self-management, improved functioning and recovery-oriented role outcomes, and reduced relapse and rehospitalization (Morrison and Barratt, 2010; Berry and Hayward, 2011; Dixon et al., 2015; Keepers et al., 2020). Figure 2 summarizes a participation-capacity care pathway model illustrating where KMT could be introduced as an adjunct to improve CBTp initiation, continuity, and between-session practice in schizophrenia spectrum disorders.

## 6 Conclusion

Given heterogeneity in schizophrenia spectrum presentations and the methodological limits common to early stage evidence (e.g., small samples, variable comparators, and inconsistent follow up), it remains clinically plausible that many individuals will show no change, only partial improvement, or improvement that is restricted to certain domains (e.g., arousal, sleep, or distress rather than core positive symptoms) with KMT. Accordingly, the magnitude, durability, and moderators of psychosis symptom change after KMT initiation remain uncertain (Longhitano et al., 2024; Rog et al., 2024). These questions should be addressed in adequately powered trials using standardized psychosis outcomes (Longhitano et al., 2024; Rog et al., 2024), prespecified responder definitions, combining quantitative measures of symptom severity and functional impairment (Keepers et al., 2020; Sawada et al., 2024), with qualitative analyses of user experiences and careful characterization of who benefits and who does not (Laurent, 2024; Sawada et al., 2024; Reznik et al., 2025).

However, symptom change in hallucinations or paranoia is not the sole clinical rationale for CBTp delivery, because CBTp targets multiple mechanisms and outcomes that remain relevant even when positive symptoms are unchanged, intermittent, or already improving (Morrison and Barratt, 2010; Berry and Hayward, 2011; Keepers et al., 2020; Wood et al., 2022). CBTp is commonly organized around reducing participation limiting factors (e.g., distress tolerance, avoidance/safety behaviors, cognitive burden, demoralization, and comorbid affective symptoms), strengthening coping and self-management skills, and supporting functional and recovery-oriented goals that extend beyond symptom counts (Morrison and Barratt, 2010; Berry and Hayward, 2011; Mueser et al., 2015). On this view, CBTp retains clear clinical utility for improving engagement, stability, and day-to-day functioning in psychosis-spectrum populations, irrespective of whether psychotic symptoms fully remit, partially remit, or persist over time (Berry and Hayward, 2011; Keepers et al., 2020).

While the current research literature supports restraint in claims about KMT as a psychosis treatment, it does potentially support a narrower and clinically actionable agenda for clinical psychology. Because CBTp benefits depend on treatment receipt, and because receipt remains limited (Wood et al., 2022, 2025) in routine services, the key question is whether KMT could reduce participation limiting distress and cognitive burden enough for more individuals to initiate, sustain, and complete CBTp, and thereby achieve the coping, stability, and recovery oriented functional gains that CBTp targets even when positive symptoms persist or fluctuate (Morrison and Barratt, 2010; Berry and Hayward, 2011; Sitko et al., 2020; Rog et al., 2024). This framing keeps the evidentiary bar for symptom change conclusions appropriately high while directing attention to the practical delivery problem that clinicians face, of how to help more people remain engaged long enough for CBTp to be delivered as intended (Dixon et al., 2015) and translated into meaningful recovery outcomes (Morrison and Barratt, 2010; Berry and Hayward, 2011).
